# The Amount of Orthodontic Force Reaching the Dental Pulp and Neuro-Vascular Bundle During Orthodontic Movements in the Intact Periodontium

**DOI:** 10.3390/medicina60122045

**Published:** 2024-12-12

**Authors:** Radu-Andrei Moga, Cristian Doru Olteanu, Ada Gabriela Delean

**Affiliations:** 1 Department of Cariology, Endodontics and Oral Pathology, School of Dental Medicine, University of Medicine and Pharmacy Iuliu Hatieganu, Str. Motilor 33, 400001 Cluj-Napoca, Romania; ada.delean@umfcluj.ro; 2 Department of Orthodontics, School of Dental Medicine, University of Medicine and Pharmacy Iuliu Hatieganu, Str. Avram Iancu 31, 400083 Cluj-Napoca, Romania

**Keywords:** dental pulp, intact periodontium, neuro-vascular bundle, numerical study, stress absorption–dissipation, orthodontic force

## Abstract

*Background and Objectives*: Most orthodontic forces are absorbed–dissipated before reaching the dental pulp and its neuro-vascular bundle (NVB); however, no data are available about their amounts. The objective of this study was to assess the amount of orthodontic force that reaches the dental pulp and its NVB during orthodontic movements in a healthy periodontium. *Materials and Methods*: This study involved the second lower premolars of nine patients and 180 numerical simulations. Five orthodontic movements (intrusion, extrusion, rotation, translation, and tipping) under 0.5 N/5 KPa and 4 N/40 KPa were assessed. The numerical methods included only two failure criteria suitable for dental tissue (of ductile resemblance): Von Mises (VM) (overall, homogenous) and Tresca (shear, non-homogenous). *Results*: Both forces displayed a similar color-coded stress display for the two methods. The Tresca quantitative results were 1.11 times higher than the VM but lower than the maximum physiological hydrostatic circulatory pressure. The biomechanical behavior of the pulp and NVB showed that, in the intact periodontium, the NVB-induced stress was 5.7 higher than in the pulp. Quantitatively, the rotation movement seemed to be the most stressful for the NVB, closely followed by intrusion and extrusion. For the dental pulp, rotation remained the most stressful, closely followed by tipping and translation. Tissue deformations were visible for NVB areas during intrusion and extrusion. The dental pulp showed pulpal stresses under translation and rotation. The numerical simulations with the two methods showed that, in the intact periodontium, only a small amount of the initial orthodontic load produced effects in the NVB and dental pulp. Only about 2.85% of the initial orthodontic load of 40 KPa/4 N applied at the bracket level induced stresses in the NVB, while the dental pulp was reached by 0.5% of the applied force. A similar distribution was seen at 5 KPa/0.5 N. *Conclusions*: The absorption–dissipation ability of the dental tissue varies between 97.15 and 99.98%.

## 1. Introduction

The amount of orthodontic force applied during treatment can induce various local circulatory disturbances in the dental pulp and its neuro-vascular bundle (NVB) [1,2,3,4,5]. Their severity and intensity depend on the amount of the applied orthodontic force that reaches these tissue structures [1,6,7]. However, the same local circulatory disturbances also trigger orthodontic movement [8,9,10]. It must be emphasized that much of the orthodontic load is absorbed and dissipated by the tooth and periodontal ligament before reaching the pulp and NVB [1,10,11]. Nevertheless, this ability has not yet been individually assessed [12,13].

The local physiological maximum hydrostatic pressure (MHP) present at this level is about 16–22 KPa (about 80% of the systolic pressure) [1,2,3,4,5]. If this amount is exceeded, local ischemia is induced, which triggers the local remodeling processes related to orthodontic movement. The time and intensity highly influence the outcomes of these circulatory disturbances [1,6,7,14,15,16]. In healthy intact tissue, usually under light and moderate orthodontic forces, there is limited ischemia, with no further consequences due to the physiological tissue’s ability to sustain, limit, and recover after tissular damage [1,10,11,17,18,19]. However, if there has been previous occlusal trauma with a strong impact on the NVB [20,21,22] and/or coronal pulpal injuries due to direct and indirect pulp capping [1,10,11,23,24,25,26], this ability to sustain damage is severely diminished and ischemia is triggered, closely followed by local tissular necrosis and further degenerative and resorptive processes [1,10,11,23,24,25,26,27,28,29,30,31,32,33,34]. These previously mentioned injuries and trauma [16,17,19,32] are rarely clinically visible and usually pass unnoticed [20,23]. Their effects become clinically visible only after the irreversible pathological processes start [20,23,32]. Thus, the same amount of orthodontic force can be safely applied in healthy intact tissue or induce severe effects in other tissue types with a clinically healthy appearance [16,17,18,19,28,29,30,31,33,34]. Thus, there is a need to investigate the individual biomechanical behavior of the pulp and its NVB under orthodontic forces and movements in healthy intact tissue. It must be emphasized that no similar studies can be found except our previous ones [12,13,35,36,37].

The tissular biomechanical behavior of dental tissue can be assessed only through numerical studies [12,13,35,36,37,38]. No such studies can be found, mainly due to the complexity of the anatomical model’s reconstructions [12,13,35,36,37]. However, multiple numerical studies of the periodontal ligament– and bone–implant interface are available in the current research flow, reporting various results due to accuracy issues [12,13,35,36,37]. It must be emphasized that the periodontal ligament anatomically holds the NVB structure in its apical third, with a strong impact on its biomechanical behavior [12,13,35,36,37].

Numerical studies derived from the engineering field are widely used and recognized for their remarkable accuracy [12,13,35,36,37,38]. In the dental field, they were introduced in the past decade but have provided multiple contradictory reports with accuracy issues due to the misunderstanding of the principles underlying numerical studies [12,13,35,36,37,38]. In our previous studies, our team identified the main accuracy issues and provided methodological improvements, reporting accurate results [12,13,35,36,37]. These were related to the use of brittle and hydrostatic [2,3,4,5,16,17,18,19,33,39,40,41,42,43,44,45,46,47,48,49,50,51,52,53,54,55] failure criteria for ductile [12,13,35,36,37] dental tissue, low-accuracy tissular models, boundary assumptions that are inconsistent with the clinical reality, and a lack of correlation with the MHP. However, all data needed to overcome these above-mentioned problems are also available in the engineering field [12,13,35,36,37,38].

The current numerical dental studies [2,3,4,5,16,17,18,19,33,39,40,41,42,43,44,45,46,47,48,49,50,51,52,53,54,55] provide contradictory results on the safest amount of force to be applied in an intact periodontium during orthodontic treatment, ranging between 0.2 and 6 N, while the most stressful movements reported are rotation, intrusion, and translation. Moreover, recent reports are conflicting regarding the poor quality of multiple in vivo studies [9,14,33] due to methodology issues. Thus, there is a need for newer numerical studies to assess the individual biomechanical behavior of dental tissue, following the methodological requirements to report correct results.

The objective of this study was to assess the amount of orthodontic force applied at the bracket level that produces effects at the NVB and dental pulp levels during five orthodontic movements performed in an intact periodontium.

## 2. Materials and Methods

Our study was part of a larger, stepwise research work, with clinical protocol 158/02.04.2018, continuing to study the effects of orthodontic forces and movements on dental tissue during periodontal breakdown [10,11,12,13,35,36,37].

This study focused on the biomechanical behavior of the pulp and NVB in the intact periodontium, focusing on the second lower premolars of nine patients, namely four males and five females with a mean age of 29.81 ± 1.45 years, totaling 180 numerical simulations.

The inclusion criteria consisted of a complete mandibular dental arch in the region of interest (i.e., the two premolars and first lower molar), no malposition, intact healthy teeth, 1–2 mm bone loss in the region of interest, a healthy periodontium, orthodontic treatment indication, and good oral hygiene.

The exclusion criteria consisted of a particular root geometry, an abnormal crown shape, abnormal root surface defects, radiologically visible bone defects, an abnormal pulp chamber and root canals, more than 2–3 mm bone loss, and poor oral hygiene after acceptance.

By using a CBCT scan (ProMax 3DS, Planmeca, Helsinki, Finland; voxel size of 0.075 mm), the lower premolar regions of the nine patients were investigated.

**Figure 1:**
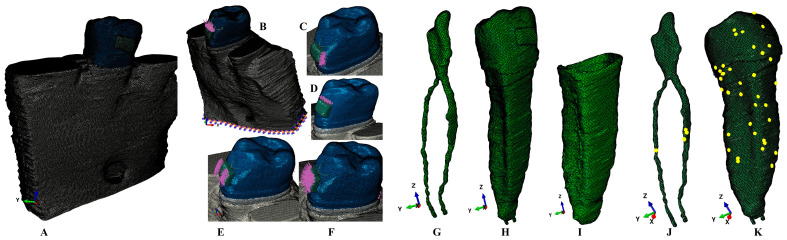
Mesh model of one of the nine 3D models: (**A**) second lower-right premolar model with intact periodontium, (**B**) applied vectors for intrusion, (**C**) translation vector, (**D**) extrusion vector, (**E**) rotation vector, (**F**) tipping vector, (**G**) dental pulp and NVB, (**H**) premolar with dental pulp and NVB, (**I**) periodontal ligament, (**J**) dental pulp mesh element warnings, (**K**) second lower premolar mesh element warnings.

The CBCT scans were then loaded into AMIRA 5.4.0 (Visage Imaging Inc., Andover, MA, USA), an image reconstruction software program, where each individual tissular component was identified and manually reconstructed. Thus, the enamel, dentine, dental pulp, periodontal ligament (PDL), neuro-vascular bundle (NVB), and trabecular and cortical bone were identified and reconstructed (Figure 1) into 3D models. The alveolar sockets of the first lower molar and premolar were filled with trabecular and cortical bone. The cementum could not be separated from the dentine; thus, it was reconstructed as dentine due to their similar physical properties; see Table 1. The PDL was reconstructed with the anatomical variable thickness of 0.15–0.225 mm. The missing bone and periodontal ligament were reconstructed to obtain 3D models with an intact periodontium. The base of a stainless-steel bracket was reconstructed on the enamel of the crown.

The results provided by AMIRA 5.4.0 were nine intact periodontium mesh models with 5.06–6.05 million C3D4 tetrahedral elements, 0.97–1.07 million nodes, and a global element size of 0.08–0.116 mm. No element errors were found in the mesh models. However, a few element warnings were present in non-essential areas, while the essential areas were continuous. In the pulp and NVB of one of the models, four element warnings were found, amounting to 0.0158% of the total of 25,252 elements. In the same mesh model, the tooth mesh displayed thirty-nine element warnings, amounting to 0.00589% of the total of 661,137 elements. All internal algorithms checks were successfully passed, and the mesh was prepared to be imported into the numerical analysis software.

ABAQUS 6.13-1(Dassault Systèmes Simulia Corp., Maastricht, The Netherlands) was used for the numerical study. The simulated orthodontic movements consisted of intrusion, extrusion, rotation, tipping, and translation. The orthodontic forces were 0.5 N/5 KPa and 4 N/40 KPa. The employed failure criteria were those for ductile-like materials, namely Von Mises (overall, homogenous) and Tresca (shear, non-homogenous), which are suitable for dental tissue. As boundary assumptions, isotropy, homogeneity/non-homogeneity, linear elasticity, perfectly bonded interfaces, and zero displacements in the bases of the models were assumed (similarly to most numerical studies).

The numerical simulations’ results were displayed as color-coded projections of various intensities and consisted of red–orange high, yellow–green moderate, and blue low shades. The mean average NVB and pulpal stress for each movement and failure criterion were registered. The amount of stress was compared with the local maximum physiological hydrostatic pressure of 16–22 KPa. The results were then correlated with known clinical biomechanical behavior and other numerical studies.

## 3. Results

**Figure 2:**
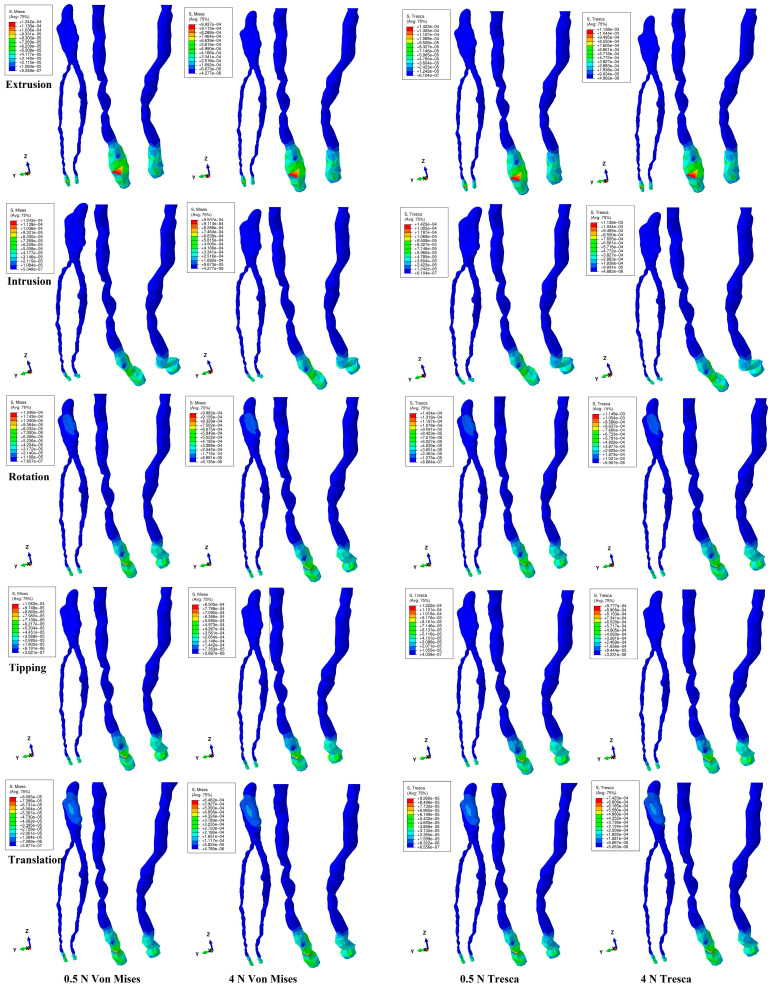
Comparative stress distribution in one of the nine 3D intact periodontium models using the Von Mises and Tresca numerical methods, for 0.5 and 4 N applied forces and five movements.

The numerical simulations with the two methods showed that, in the intact periodontium, only a small amount of the initial orthodontic load produced effects in the NVB and the dental pulp (Figure 2 and Table 2). Both numerical methods displayed similar qualitative results, while the quantitative ones were slightly smaller for Von Mises when compared with Tresca. The quantitative difference between the Tresca and Von Mises results, for both forces, was an average of 1.11 times (1.17 times for NVB and 1.05 times for pulp), falling within the scientifically specified average interval of 1.15 times (around 10%). In the intact periodontium, the orthodontic forces induced NVB stresses that were 5.7 times higher than in the dental pulp.

The orthodontic loads produced visible effects at the NVB level and almost none at the pulp level (except in the translation movement). Thus, from the initial 4 N/40 KPa of orthodontic load, only around 1 KPa was manifested as stress at the NVB level. Both methods similarly showed the rotation, followed by the extrusion and intrusion, displaying stresses of around 1 KPa at the NVB level, i.e., about 2.85% of the initial orthodontic load of 40 KPa applied at the bracket level. This implies that the remaining 97.15% of the orthodontic load was absorbed and dissipated by the other dental components—the dentine, enamel, stainless-steel bracket base, and PDL—before arriving at the NVB. Since this amount was lower than the physiological maximum hydrostatic circulatory pressure of 16–22 KPa at this level, a load of 40 KPa seems to be safe for the NVB and pulp. During the rotation movement, the pulp received only 0.17–0.20 KPa of the initial 40 KPa applied at the bracket level, i.e., about 0.5% of the applied load, while the remaining 99.5% seemed to be absorbed and dissipated by the enamel, dentine, and stainless-steel bracket.

The 0.5 N/5 KPa load produced similar stress results, with 2.8% of the initial load producing effects at the NVB level and 0.02% at the pulp level, showing a tissular absorption–dissipation capacity of 97.2–99.98% (by the dentine, enamel, stainless-steel bracket base, and PDL) of the initial applied amount of orthodontic force.

Both the Tresca and Von Mises methods showed similar color-coded stress displays for both loads. The NVB tissular deformations were more visible for intrusion and extrusion movements. Translation and rotation movements were the only ones to display coronal pulp stress (Figure 2), which was higher on the proximal sides (mesial and distal) and lower on the vestibular one. These amounts of stress, due to being lower than the maximum physiological hydrostatic circulatory pressure, had no impact on the healthy and intact pulp and NVB (since this tissue has a natural ability to sustain damage). Quantitatively, the rotation movement induced the highest stresses in the NVB, closely followed by intrusion and extrusion, appearing to be the most stressful for the NVB. For the dental pulp, rotation remained the most stressful, closely followed by tipping and translation.

## 4. Discussion

This analysis aimed to assess, in the intact periodontium, the amount of load reaching the dental pulp and its NVB when compared with the initial applied orthodontic load. To obtain correct results, only two numerical methods are suitable for these tissue types [12,35,36,37]. The individual study of the pulp and NVB is possible only through numerical methods [12,35,36,37,38]. Five orthodontic movements and two loads (0.5 N—light and 4 N—moderate to large) were used in a total of 180 simulations. We must emphasize that our study is the first to assess this issue and the benefits of this approach. No other studies, except our earlier ones [12,35,36], related to these issues can be found in the current research flow. Both used methods were reported to be the most correct and suitable for dental tissue, being especially designed for ductile resemblance in homogenous and non-homogenous materials [12,35,36]. Dental tissue has been reported to have ductile resemblance but with a certain brittle flow mode [12,13,35,36,37].

Both the Von Mises and Tresca methods reported similar color-coded stress display projections in the pulp and NVB, with higher tissular deformation visible in the NVB during the five orthodontic movements and two forces, in line with our earlier reports [12,35,36]. The quantitative results displayed a similar pattern, with the amounts of stress being lower than the physiological maximum hydrostatic pressure of 16–22 KPa, and being 5.7 times higher in the NVB than in the pulp, in line with earlier reports [12,35,36]. From the total amount of the applied load, only around 2.8% induced stress in the NVB and 0.02–0.5% in the dental pulp. Thus, 97.2–99.9% of the load was absorbed and dissipated by the enamel, dentine, and stainless-steel bracket, in line with earlier reports [12,13,35,36]. All previously mentioned tissue types and materials are considered to have ductile biomechanical behavior [12,13,35,36,37]. Only two studies [12,13] related to this subject can be found in the current research flow, both belonging to our team. In an earlier report [12], we found an absorption–dissipation capacity of 40–93% for the dentine, 40–65% for the enamel, and 16% for the stainless-steel bracket, in line with the present work. The same study [12] reported that, from the total amount of the orthodontic load, only a small percentage reached the PDL (0.3–8.4%), NVB (0.2–0.7%), and dental pulp (0.02–0.12%), also in agreement with the present work. The other study [13] reported the tooth structure’s absorption–dissipation capacity to be 85% of the stress before reaching the sensitive circulatory tissue, with 86.66–97.5% of the stress being dissipated before reaching the PDL, 98% before reaching the NVB, and 99.6–99.94% before reaching the pulp, in agreement with the present work. Ductility is the ability of a material to deform under a load without breaking and to recover its original form when the load is removed [37]. We must emphasize that, under the orthodontic forces and during the movements, the displacements and tissular deformations were extremely small when compared with those described by physical mechanics in the engineering field [13,37]. Forces of 0.5 and 4 N are extremely small from an engineering point of view [13,37].

Contradictory results were obtained when using other numerical methods (maximum and minimum principle—brittle-like materials and hydrostatic pressure—liquid/gas), since these failure criteria were especially designed in the engineering field for the study of a specific type of biomechanical behavior. Earlier studies by our group reported these methods to be less exact than the Tresca and Von Mises methods [12,35,36,37]. Since no similar studies could be found, the only possible correlations could be performed with PDL numerical studies (since, anatomically, the NVB is held by the periodontal ligament in the apical third). However, these PDL numerical studies mostly used the maximum and minimum principle, as well as hydrostatic pressure criteria (proven by our earlier studies to suffer from accuracy issues), reporting results that varied from one study to another or simply contradicting the clinical knowledge (but without any discussions about these issues). In particular, Wu et al. employed hydrostatic pressure [2,3,48] when assessing the optimal orthodontic force in premolar rotation. They reported 2.1–2.9 N rotation to be optimal, showing amounts of stress lower than the MHP and finding only PDL apical third stress and no cervical third stress, which contradicts the existing clinical knowledge. Hofman et al. [4,5] reported values of 80 KPa for 1 N of intrusion and around 40 KPa for 3–6 N lingual torque, implying degenerative and resorptive processes, which clinically is incorrect. Even new studies persist in adopting the same approach of employing brittle and hydrostatic liquid criteria for the study of dental tissue with a ductile-like resemblance, without considering the abovementioned issues [49,50,51,52].

From a biomechanical point of view, during the orthodontic movements, intrusion and extrusion displayed higher NVB tissular deformations, in agreement with Minch et al. [7] and Hofman et al. [4,5]. Translation (barely visible in rotation) displayed coronal pulp stress areas, found on the mesial and distal sides (higher) as well as on the vestibular side (lower). In both the NVB and dental pulp, rotation displayed the highest amounts of stress, in agreement with Wu et al.’s numerical reports [2,3,48]. However, the abovementioned factors seemed not to induce any effects in the healthy intact tissue, in agreement with other reports [15]. Regarding the stress absorption–distribution pattern, 4 N/40 KPa of rotation, tipping, and translation displayed values of 0.13–0.20 Kpa in the coronal pulp (0.5% of the applied load), showing an absorption–dissipation capacity of 99.5% for the dental tissue surrounding the pulp, i.e., the enamel and dentine (the bracket also might have a contribution), in line with previous reports [12,13].

The same amount of 40 KPa during rotation, tipping, extrusion, and intrusion induced 0.93–1.14 Kpa in the NVB (2.8% of the applied load), showing an absorption–dissipation capacity of 97.5% in the surrounding dental tissue, i.e., the PDL, dentine, and enamel (the bracket might also contribute), in line with other reports [12,13]. This percentage difference between the pulp and NVB is probably linked to the PDL component, since the neuro-vascular bundle is held in the apical third of the periodontal ligament, suffering from important tissular deformations during movement [12,13]. It must be emphasized that the orthodontic movements and tissular deformations are extremely small (as herein reported), with no induced ischemic risks due to their natural ability to adapt to trauma [1,6,7,16,19,25,26,27,28,29,30,31], as reported in our previous works [12,13,35,36,37] and other reports [2,3,4,5,48,49,50,51,52]. It must also be emphasized that the previously mentioned findings refer to intact healthy tissue with an intact ability to adapt to ischemia and to prevent local resorptive and degenerative processes. If these tissue types were previously traumatized and/or injured, as is clinically possible through occlusal trauma [20,21] and direct or indirect pulpal capping [1,10,11,23,24,26,27,32], their functional ability to adapt would be diminished due to internal pathological and histomorphological changes [14,29,30,32,34]. Thus, if, in the intact healthy periodontium, a light or moderate (as herein proven) orthodontic force induces no ischemic risks when triggering a movement, the same force could pose ischemic risks in the presence of a traumatized and/or injured NVB and dental pulp [8,9,10,16,22,24,25,26,32], and the used amount must be considered with care.

To provide accurate results, the numerical methods must follow the engineering field’s requirements: failure criteria must be selected on the basis of the analyzed type of material, with boundary assumptions describing the behavioral conditions and accurate and detailed 3D models [12,35,36,37,38]. Nevertheless, most of the numerical dental studies found in the current research field do not acknowledge the abovementioned aspects [4,5,16,17,18,19,33,39,40,41,42,43,44,45,46,47], with their reports showing various accuracy issues [12,35,36,37,38].

The individual study of the dental pulp and NVB can be achieved only through numerical studies. For dental studies, the only suitable failure criteria are the Von Mises and Tresca ones, since they are the only ones to be specifically designed for ductile-like materials such as dental tissue [12,35,36,37]. All other methods are designed for brittle and liquid-like substances and provide less accurate results when employed, as previously reported [12,35,36,37,38].

The boundary assumptions for dental studies are related to homogeneity/non-homogeneity, isotropy–anisotropy, and linear/non-linear elasticity, as well as the amount of applied load [12,35,36,37,38]. Natural tissue is non-homogenous, anisotropic, and non-linear elastic. Numerical studies cannot completely reproduce the clinical reality, but they are the only possible option to individually study these small tissue types [12,35,36,37]. The applied load is extremely important when describing the tissular biomechanical behavior, since, in biomechanical physics, under small forces, all materials show linear elasticity [12,35,36,37]. It must be emphasized that forces of up to 4 N are considered extremely small from an engineering point of view [12,35,36,37]. Moreover, there are multiple comparative reports assessing various amounts of force ranging from 0.2 to 4.8 N, as conducted by our team, reporting similar stress distribution areas for these forces, confirming the view of the engineering field [12,35,36,37]. These abovementioned findings are also supported by the clinically identified extremely small tissular deformations and displacements [15]. Thus, under these circumstances, the linear elasticity assumption used in previous dental studies is correct [2,3,4,5,16,17,18,19,33,39,40,41,42,43,44,45,46,47,48,49,50,51,52]. Zhang et al. [53] reported that, when using the abovementioned assumed boundary conditions, the results were closer to those of clinical and animal studies. In dental studies, only the PDL is assessed as non-linear or linear, reporting various results, while all other components are seen as linear. Toms et al. [39] investigated 1 N of extrusion in an intact PDL and reported higher amounts for linear than non-linear when using the maximum and minimum principle method. In contrast, Hemanth et al. [40,41] assessed these differences in an intact periodontium, reporting, for the maxillary central incisor PDL, values ranging between 20 and 50% (148,097 elements and 239,666 nodes). Nevertheless, they considered 0.2–1 N for intrusion and tipping but used the maximum and minimum principle method (specifically designed for brittle materials) to describe the biomechanical behavior of ductile-like tissue.

The homogeneity and non-homogeneity issue is also approached herein, since the Von Mises method assesses homogenous materials while Tresca considers non-homogenous ones [12,35,36,37]. The average difference between them in scientific engineering is around 1.15 times (about a 10–15% difference), with Tresca being higher than VM, with the present results (1.11 times/11% difference) falling within the specified interval, in line with our previous reports [12,35,36,37]. This issue has not been addressed in existing numerical studies [2,3,4,5,16,17,18,19,33,39,40,41,42,43,44,45,46,47,48,49,50,51,52].

In our study, rotation, closely followed by tipping, extrusion, and intrusion, was more stressful for the NVB, while, for the dental pulp, rotation remained the most stressful, followed by tipping and translation. Our findings are in line with Wu et al. [2,3,48] and Hofman et al. [4,5], despite their use of brittle-like failure criteria for ductile dental tissue. However, there are many other numerical studies reporting various contradictory results regarding the most stressful orthodontic movement, although none of them includes a comparison of the five movements or employs ductile-like materials [44,54,55].

The levels of detail in the 3D model are of extreme importance, especially when studying small and complex dental tissue [12,35,36,37,38]. Most numerical studies have employed low levels of detail in anatomically idealized models, with a reduced number of elements and nodes and a larger global element size, strongly affecting the accuracy of the results. Thus, most studies [2,3,4,5,16,17,18,19,33,39,40,41,42,43,44,45,46,47,48,49,50,51,52] have employed low-anatomical-accuracy 3D models (without NVB or dental pulp tissular reconstructions), with a large global element size of up to 1.2 mm and a small number of elements/nodes, e.g., 1674/5205-23563/32812-1.67 million. The present study employed models with a reduced global element size of 0.08–0.116 mm and a large number of elements/nodes of 5.06–6.05 million/0.97–1.07 million, better meeting the anatomical accuracy criteria.

The sample size is specific to the numerical study due to the great variability in the experimental conditions, leading to different results [12,35,36,37,38]. Most dental numerical studies have used a sample size of one [2,3,4,5,16,17,18,19,33,39,40,41,42,43,44,45,46,47,48,49,50,51,52,54]. To enhance our results’ accuracy, we used a sample size of nine.

The limitation of the numerical method lies in not being able to entirely reproduce the clinical conditions. In the clinical setting, there are often associations among movements and not a pure movement, as in the present analysis. This could mean that the clinical amounts of stress are in fact even lower than those identified, but with no impact on their accuracy. Moreover, this is the only available possibility for individual tissular studies. To ensure accuracy, the method needs anatomically correct 3D models (which are extremely difficult to reconstruct), and it is necessary to employ failure criteria that are suitable for the analyzed material, while the boundary assumptions should agree with the clinical conditions (as previously discussed). Any deviations from these mandatory requirements will lead to incorrect results and can be considered as limiting the method. Thus, the reduced number of numerical studies meeting the abovementioned criteria, as well as reports regarding the poor quality of multiple in vivo studies [9,14,33] used for indirect validation, emphasizes the need for more numerical studies in order complete the data. Moreover, since the PDL is reported to play a key role in the load’s absorption–dissipation during tooth biomechanics, further numerical simulations are needed to assess this ability.

## 5. Conclusions

1. The numerical simulations with the two methods showed that, in the intact periodontium, only a small amount of the initial orthodontic load produced effects in the NVB and dental pulp.
2. Only about 2.85% of the initial orthodontic load of 40 KPa/4 N applied at the bracket level induced stresses in the NVB, while this was 0.5% for the dental pulp. A similar distribution was seen for the force of 5 KPa/0.5 N.
3. The absorption–dissipation ability of the dental tissue varied between 97.15 and 99.98%.
4. Both forces displayed quantitative stress amounts that were lower than the maximum physiological hydrostatic circulatory pressure, appearing not to produce harmful effects on the healthy and intact pulp and NVB.
5. Quantitatively, the rotation movement seems the most stressful for the NVB, closely followed by intrusion and extrusion. For the dental pulp, rotation remained the most stressful, closely followed by tipping and translation.
6. Tissular deformations were visible for the NVB area during intrusion and extrusion. The dental pulp showed pulpal stresses under translation and rotation.
7. Clinically, it appears that up to 4 N of applied force is safe for the NVB and dental pulp, since only 0.5–2.85% of the load reaches these tissue types.

### Practical Implications

Numerical studies of the dental pulp and NVB are scarce; only a few are available, with most of them belonging to our research team. This is the first numerical study to directly investigate the amount of stress reaching these small dental tissue types (about 0.02–2.85%) when compared with the total amount of applied orthodontic force. This study, conducted on the intact periodontium and healthy tissue, proved that both light and moderate orthodontic forces have similar quantitative tissular absorption–dissipation patterns, with an absorption rate of 97.15–99.98%. Moreover, forces of 0.5 and 4 N displayed similar biomechanical behavior, with tissular deformations of the NVB and coronal pulpal stress areas induced by translation and rotation movements, with no impact on the healthy intact tissue but with importance for previously traumatized tissue. Moreover, using the only two numerical methods suitable for the dental tissue, our study proved that, up to 4 N, there is no NVB stress exceeding the local physiological maximum hydrostatic pressure; this is of importance for the clinical phase of orthodontic treatment. For researchers, this study provides information on how to conduct an accurate numerical dental study, as well as a clear picture of the problems related to the boundary assumptions and the method selection.

## Figures and Tables

**Table 1:** Physical properties of materials.

Material	Young’s Modulus, E (GPa)	Poisson Ratio, υ	Refs.
Enamel	80	0.33	[10,11,12,13,35,36,37]
Dentin/cementum	18.6	0.31	[10,11,12,13,35,36,37]
Pulp and NVB	0.0021	0.45	[10,11,12,13,35,36,37]
PDL	0.0667	0.49	[10,11,12,13,35,36,37]
Cortical bone	14.5	0.323	[10,11,12,13,35,36,37]
Trabecular bone	1.37	0.3	[10,11,12,13,35,36,37]
Stainless-streel bracket (Cr-Co)	218	0.33	[10,11,12,13,35,36,37]

**Table 2:** Maximum stress average values (KPa) produced by 0.5 N and 4 N in the NVB and coronal pulp by the two methods.

Resorption (mm)			Extrusion	Intrusion	Rotation	Tipping	Translation
4 N/40 KPa	Tresca	NVB	0.96	0.93	1.14	0.98	0.74
		c	0.10	0.10	0.20	0.16	0.13
	VM	NVB	0.75	0.75	1.00	0.85	0.64
		c	0.09	0.09	0.17	0.14	0.16
0.5 N/5 KPa	Tresca	NVB	0.12	0.12	0.14	0.12	0.09
		c	0.01	0.01	0.02	0.02	0.02
	VM	NVB	0.09	0.09	0.13	0.11	0.08
		c	0.01	0.01	0.02	0.02	0.02

NVB—neuro-vascular bundle, c—coronal pulp.

## Data Availability

The original contributions presented in this study are included in the article. Further inquiries can be directed to the corresponding authors.
